# Phytochemical, Cytoprotective Profiling, and Anti-Inflammatory Potential of *Colchicum luteum* in Rheumatoid Arthritis: An Experimental and Simulation Study

**DOI:** 10.3390/nu16234020

**Published:** 2024-11-24

**Authors:** Huda Abbasi, Maria Sharif, Peter John, Attya Bhatti, Muhammad Qasim Hayat, Qaisar Mansoor

**Affiliations:** 1 Atta ur Rahman School of Applied Biosciences, National University of Sciences and Technology, Sector H-12, Islamabad 44000, Pakistan; habbasi.phdabs14asab@asab.nust.edu.pk (H.A.); msharif.phdabs22asab@student.nust.edu.pk (M.S.); attyabhatti@asab.nust.edu.pk (A.B.); m.qasim@asab.nust.edu.pk (M.Q.H.); 2 Institute of Biomedical and Genetic Engineering (IBGE), Sector G-9/4, Islamabad 44000, Pakistan; qmibge@gmail.com

**Keywords:** plant extract, anti-inflammatory, NSAIDs, acute toxicity, phytochemicals, bioactive compound compounds, simulations

## Abstract

Background: Rheumatoid arthritis (RA) is a chronic autoimmune disorder characterized by severe pain, inflammation, and joint deformity. Currently, it affects 1% of the population, with a projection to exceed 23 million cases by 2030. Despite significant advancements, non-steroidal anti-inflammatory drugs (NSAIDs), the first line of treatment, are associated with a range of adverse effects. Consequently, plant-based derivatives are being utilized as an effective alternative. This study evaluates the anti-inflammatory and safety profile of *Colchicum luteum* hydroethanolic extract (CLHE) in comparison to NSAIDs, with a focus on COX-2 and TNFα inhibition. Methods: CLHE potential was evaluated by phytochemical screening and in vitro bioactivity assays. Toxicity profile was conducted in Human Colon Epithelial Cells (HCEC) and Balb/c mice. Anti-inflammatory potential was explored in a collagen-induced arthritic (CIA) mice model. Bioactive compounds were identified computationally from GCMS data and subjected to docking and simulation studies against COX2 and TNFα. Results: CLHE demonstrated significant antioxidant (IC-50 = 6.78 µg/mL) and anti-inflammatory (IC-50 = 97.39 µg/mL) activity. It maintained 50% cell viability at 78.5 μg/µL in HCEC cells and exhibited no toxicity at a dose of 5000 mg/kg in mice. In the CIA model, CLHE significantly reduced paw swelling, arthritic scoring, C-reactive protein levels, and spleen indices, outperforming ibuprofen. Expression analysis confirmed the downregulation of COX-2, TNFα, and MMP-9. Histopathological analysis indicated the superior efficacy of CLHE compared to ibuprofen in reducing inflammation, synovial hyperplasia, and bone erosion. Computational studies identified compound-15 (CL15), (4-(4,7-dimethoxy-1,3-benzodioxol-5-yl)-2-oxo pyrrolidine-3-carboxylic acid), a non-toxic compound with strong binding affinities to COX-2 (−12.9 KJ/mol), and TNF-α (−5.8 KJ/mol). Conclusions: The findings suggest the potential of *Colchicum luteum* as a safer, anti-inflammatory, and multi-targeted alternative to NSAIDs for RA treatment.

## 1. Introduction

Rheumatoid arthritis (RA) is a progressive chronic disorder affecting bones and cartilage, leading to joint deformity and loss of function. It impacts 1% of the global population and ranks among the top 10 causes of disability worldwide. Furthermore, the trends of rheumatic diseases in Pakistan have shown an increase in prevalence, deaths, and disability-adjusted life years (DALYs) over the past 30 years, emphasizing the growing burden of rheumatic conditions in the country [1]. The onset and progression of RA are influenced by genetic predisposition, environmental factors, and lifestyle choices. The disabilities caused by chronic RA not only impair individual functioning and productivity but also place a significant economic burden on healthcare systems. Effective management strategies for RA involve early clinical diagnosis, lifestyle modifications, patient education, and advancements in therapeutic interventions [2]. Current treatments for inflammation in rheumatoid arthritis (RA) include disease-modifying antirheumatic drugs (DMARDs) and non-steroidal anti-inflammatory drugs (NSAIDs). However, both drug classes are associated with significant adverse effects, such as gastric perforation, renal and liver damage, and pancytopenia, which limit their long-term use in chronic illness patients [3]. Approximately 20 to 40% of patients experience NSAID-induced abdominal pain and diarrhea, and 15% of long-term users develop gastrointestinal ulcers and perforations, with 67% showing elevated bilirubin levels (>2.5 mg/dL). In the past decade, 0.01% of hospitalizations due to hepatotoxicity have been linked to NSAIDs [4]. Developing novel therapeutic agents involves a multi-step screening process to identify pharmacologically active compounds that inhibit biological targets while minimizing harm to healthy cells. Identifying therapeutic targets is crucial in drug discovery, and more than 1200 receptors and enzymes have been explored as potential targets for RA [5].

**Figure 1:**
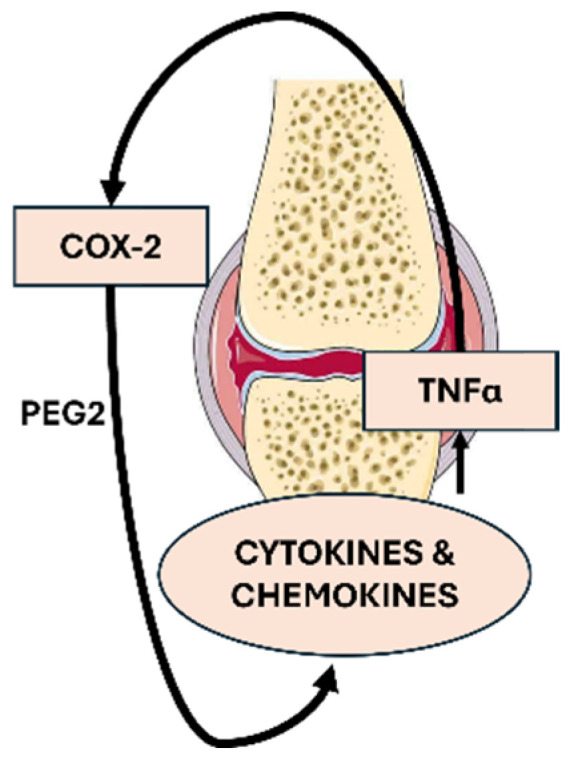
Crosstalk interaction between COX-2 and TNFα in a rheumatoid arthritis joint. COX-2 leads to TNFα activation through PGE_2_ stimulation, while TNFα simultaneously enhances COX-2 expression, creating a feedback loop that amplifies inflammation.

Cyclooxygenase-2 (COX-2), an inducible enzyme and the primary source of prostaglandins, is considered pathologic due to its role in mediating pain and inflammation [6]. Tumor necrosis factor-alpha (TNFα) is a key inflammatory cytokine that orchestrates the immune response in chronic inflammatory disorders such as RA [7]. Both COX-2 and TNFα are central to the initiation, progression, and inflammation seen in RA, with evidence suggesting that TNFα can induce COX-2 expression and vice versa (Figure 1) [8]. This highlights the importance of targeting these molecules for therapeutic inhibition.

Plant-derived compounds, known for their diverse biological activities and unique chemical structures, are being investigated as treatments for chronic diseases. Phyto-derivatives from traditional medicinal herbs, particularly those used in Chinese medicine, are often employed as complementary therapies in managing inflammatory conditions. However, the potential toxic effects of these compounds on healthy tissues remain inadequately studied [9].

*Colchicum luteum* is a medicinal herb well-known for its anti-inflammatory and analgesic properties. *C. luteum* belongs to the Colchicaceae family, which is native to China, Pakistan, India, Himalayan regions, and regions along the Mediterranean coast. Commonly known as autumn crocus or meadow saffron, this herb is identified by its characteristic yellow-colored flowers, alternate or whorled leaves, and corms or starchy rhizomes. The aerial and root parts of *C. luteum* are mentioned in Greek, Indian, and Chinese traditional medicine for their diverse medicinal applications. The corms of *C. luteum* are extensively used to treat gastric, hematological, and rheumatic diseases. *Colchicum* species have been used in traditional Chinese medicine for their antiproliferative, anti-rheumatic, antifungal, and antipyretic characteristics, and several compounds have been identified from their extracts [10,11,12,13].

Despite its extensive use in traditional medicine for the treatment of rheumatism and gout, the efficacy and safety of *Colchicum luteum* as an individual therapeutic agent for RA remains underexplored. Previous studies have shown that the methanolic extract of *C. luteum* significantly inhibits lipoxygenase activity in vitro [11], demonstrating its potential anti-inflammatory effects. Additionally, polyherbal formulations containing *C. luteum* have been investigated in clinical trials for managing joint deformities [14]. This study aims to evaluate the toxicity and anti-inflammatory potential of *C. luteum* hydroethanolic extract by targeting key RA mediators, particularly COX-2 and TNF-α. By identifying safer bioactive constituents such as CL15, we seek to establish *C. luteum* as a viable alternative to conventional NSAIDs and biologics, offering a promising therapeutic option for the management of RA.

## 2. Materials and Methods

### 2.1. The Phytochemical Analysis and Characterization of the Extract

#### 2.1.1. Identification of *Colchicum luteum* Herb and Extract Preparation

*C. luteum* was collected from Islamabad, Pakistan (33°42′35″ N 73°5′45″ E), during the fall season, from October to November 2022, as this is its flowering season. The plant was verified by taxonomists from NUST Islamabad, Pakistan. The specimen was compared to the herbarium vouchers 26,049 (RAW), 2522 (RAW), and 8457 (RAW) and confirmed to be a *Colchicum luteum* Baker (Colchicaceae). The herbarium number for *Colchicum luteum* was HUP0001096 and the taxonomy ID was 225785. The morphological and botanical information is provided on eFolras.org. For extract preparation, the corms of *Colchicum luteum* were dried and ground into a fine powder for hydroethanolic extraction (1:1 *v*/*v*), following a previously reported method with slight modifications. The mixture was macerated in the dark for two weeks and subsequently filtered using Whatman filter paper no. 1. The filtrate was then air-dried in a biosafety cabinet for 48 h [15]. The yield of the plant extract was calculated with the following formula:

$$Yield\left(\%\right)=weight\;of\;pure\;extract/weight\;of\;dried\;plant\;part\times 100$$

#### 2.1.2. Phytochemical Screening

Phytochemical analysis of the crude extract was conducted using a previously reported protocol with slight modifications [15]. An additional step involving chloroform removal through N-hexane separation was added to reduce the color intensity of the extract. The colorimetric method was used for the qualitative identification of alkaloids, phenols, flavonoids, anthocyanins, leucoanthocyanins, tannins, phlobatannins, coumarins, terpenoids, steroids, saponins, and emodin.

#### 2.1.3. Total Phenolic and Flavonoid Content

Total phenolic content was calculated using the Folin–Ciocalteu (FC) method [16]. A mixture of FC reagent (2.5 mL), NaHCO₃ (2.5 mL), and the extract (0.5 mL) was incubated at room temperature for 30 min. Gallic acid served as the standard, and absorbance was measured at 765 nm using a UV-vis spectrophotometer. Results were expressed as milligrams of gallic acid equivalent (GAE) per gram of dry sample.

The total flavonoid content of CLHE was quantified by the aluminum chloride (AlCl_3_) method [16]. After serial dilution of the extract, 5% sodium nitrite was added and incubated for 5 min at 25 °C. Then, 10% AlCl₃ was added, followed by sodium hydroxide. A calibration curve was prepared using rutin as the standard, and absorbance was measured at 510 nm. The results were expressed as milligrams of rutin equivalent per gram of dry extract.

#### 2.1.4. Chromatographic Characterization

The dried extract was dissolved in the methanol in the ratio of 1:1, which was injected into a capillary column fused with 1,4-bis (dimethyl-siloxy) phenylene dimethyl polysiloxane (0.25 μm × 20 m × 0.25 mm). The gas chromatograph was coupled with an SH-Rxi-5Sil mass spectrometer (QP-2020, SHIMADZU (Kyoto, Japan). The system was run with 70 eV ionization energy for 38 min at 1 mL/min gas flow rate and 100 °C final oven temperature. Mass spectrometry data was verified by the NIST (2017) library.

### 2.2. Evaluation of the Biological Activity of CLHE

#### 2.2.1. Inhibition of Protein Denaturation

The protein denaturation inhibition assay was performed with albumin [17]. Albumin was dissolved in distilled water at 1:1 *w*/*v* concentration and mixed with 2.8 mL phosphate-buffered saline (pH 6.4). The extract was serially diluted (50, 100, 150, 200, and 250 µL) and added to the albumin–PBS mixture, followed by a 15 min incubation at 37 °C. The solution was heated to 70 °C for 5 min and then cooled to room temperature. The commercially prescribed anti-inflammatory agent ibuprofen was used as a control and processed similarly to CHLE. The absorbance was measured at 660 nm using a UV-vis spectrophotometer, and percentage denaturation was calculated with the following formula:

(1) $$Denaturation\;\left(\%\right)=1-\left(Absorbance\;of\;control/Absorbance\;of\;sample\right)\times 100$$

#### 2.2.2. Free Radical Scavenging Activity

Radical scavenging or antioxidant activity was established by free radical scavenging activity using DPPH. A previously reported methodology was used with slight modifications [17]. CLHE concentrations of 10, 30, 50, 70, 90, and 110 µL were tested against identical dilutions of ibuprofen. The absorbance was measured at 570 nm using a UV-vis spectrophotometer. The percentage scavenging was calculated with the following formula:

(2) $$Scavenging\;\left(\%\right)=1-\left(Absorbance\;of\;control/Absorbance\;of\;sample\right)\times 100$$

### 2.3. Experimental Animals

Female BALB/c mice (8–12 weeks old) were obtained and housed at the animal house laboratory of ASAB, NUST. Mice weighing 30–35 g were kept in metal cages. All the obtained animals, i.e., experimental and control groups, were provided with a temperature- (25 °C ± 2) and humidity-regulated, pathogen-free environment. The acclimatization period was ten days. The Institutional Review Board (IRB No. 11-2020-01/04) of ASAB, NUST approved the study protocol. All experimental methods were carried out per the standards established by the Institute of Laboratory Animal Research, Division on Earth and Life Sciences, National Institute of Health, United States (Guide for the Care and Use of Laboratory Animals). All the mice included in the study were carefully examined for any pathological anomalies; initial inspections focused on characteristics like hair and coat color as well as the lack of any tissue damage.

### 2.4. Cell Lines

The Human Colon Epithelial Cells (HCEC) were provided by the Institute of Biotechnology and Genetic Engineering (IBGE), Islamabad. The cell lines were grown in Dulbecco’s Modified Eagle Media (DMEM) (Gibco) with 10% FBS and 0.1% Penicillin/Streptomycin (Sigma Aldrich, Taufkirchen, Germany) at 35 °C in 5% CO_2_.

### 2.5. Toxicology Profile of CLHE

#### 2.5.1. In Vitro Toxicity Studies

To determine the cytotoxicity of plant extract MTT (3-(4,5-dimethyl thiazolyl-2)-2,5-diphenyltetrazolium bromide) assay (Sigma Aldrich, Germany) was performed to evaluate cell growth and viability. In a 96-well plate, 100 µL HCEC were cultured, and serially diluted CLHE extract was added followed by incubation. After 48 h, 20 µL MTT solution and 50 µL solubilization solution were mixed in by pipetting. The plate was incubated in the dark for 30 min and absorbance was measured at 570 nm using a UV-vis spectrophotometer.

#### 2.5.2. In Vivo Toxicity Studies

Acute toxicity was measured with the Enegide method in healthy, 3–6-week-old, female Balb/c mice [18]. CLHE was orally administered in three phases in gradually increasing concentrations. After each phase, the mice were subjected to a 12 h fasting period to excrete the previous dose from the system. After each administration, the mice were observed for up to 6 h for signs of acute toxicity. After 24 h, mice were sacrificed, and their blood was collected via cardiac puncture to evaluate serum bilirubin, urea, and creatinine levels.

### 2.6. Analysis of Anti-Inflammatory Activity in Arthritic Mice Model

#### 2.6.1. Collagen Induced Arthritis (CIA) Model Development

Female mice were divided into 4 groups (1: healthy control; 2: disease control; 3: CLHE-treated; and 4: ibuprofen-treated). A CIA mice model was developed by the administration of type II collagenase, Freud Adjuvant, and bovine serum albumin via transdermal injection. At the end of week 2, the paws of immunized mice were observed for signs of edema and inflammation [19]. The mice showing arthritic indexes of 3 and 4 were selected for further study. Groups 3 and 4 were given CLHE (5000 mg/kg) and ibuprofen solution (636 mg/kg), respectively, as a treatment for 10 days. The selected dose of CLHE (5000 mg/kg) was based on previous acute toxicity studies, which demonstrated no significant adverse effects at this concentration, making it suitable for evaluating the extract’s therapeutic and safety profile [20]. The ibuprofen solution (636 mg/kg) was chosen to correspond to its established therapeutic dose in animal models, allowing for a comparative analysis of anti-inflammatory effects between CLHE and a standard NSAID.

#### 2.6.2. Arthritis Index

Inflammatory edema and arthritic index were used as markers of localized inflammation. Edema was assessed via paw volume measured using a Vernier caliper (Mitutoyo, Aurora, IL, USA). The arthritis index was measured by observing the paws of groups 1–4 and graded according to the digital arthritic index [19].

#### 2.6.3. Spleen Indices

For spleen indices, the weight of the spleen was divided by the body weight of the mice.

#### 2.6.4. Serum Antibody Analysis

C-reactive protein (CRP) was used as a marker for systemic inflammation. Mouse blood was drawn using cardiac puncture and collected in EDTA tubes. The test was performed by the ASAB Diagnostic Lab (NUST, Islamabad, Pakistan) by employing commercial ELISA kits (Elabscience) according to the manufacturer’s protocol. The levels of antibodies were examined to determine the extent of the inflammation caused by arthritis and the efficacy of the extracts in alleviating these levels.

#### 2.6.5. Quantitative Real-Time PCR Analysis

Gene expression was carried out via qPCR (Applied Biosystems, Thermo Fischer Scientific, Waltham, MA, USA). GAPDH was used as the housekeeping gene, and the relative expression of TNFα, COX-2, and MMP-9 were analyzed and recorded as fold change. The RNA was isolated by the TRIzol (Thermo Fischer Scientific, USA) method, and the cDNA was synthesized according to previously reported protocols [19].

#### 2.6.6. Histological Analysis

Hematoxylin and eosin (H&E) staining was employed for the histopathology analysis of the joints. The paw and tarsal joints were collected and stored in a 10% Formalin solution. The specimens were prepared according to the previously reported protocol. Samples were observed under the light microscope at 10× and 40× resolution. The parameters of inflammation, membrane infiltration, and bone erosion were studied and scored [19].

### 2.7. In Silico Analysis of C. luteum Bioactive Compound

#### 2.7.1. ADMET Screening of Bioactive Compounds

Canonical SMILES (Simplified Molecular Input Line Entry System) of GC–MS data and the structure of all the phyto-compounds were retrieved from the PubChem database. The bioactive compounds were shortlisted based on physicochemical parameters of molecular mass, blood–brain barrier permeability, and druggability using SwissADME (2017) and ADMET Lab 3.0 [21].

#### 2.7.2. Toxicology Profiling of Shortlisted Compounds

The virtual lab ProTox 3.0 was used to predict hepatotoxicity, immunotoxicity, cytotoxicity, and lethal dose (LD_50_). The toxicity levels were used to categorize shortlisted compounds into toxicological classes I, II (fatal), III (toxic), IV (harmful), and V (non-toxic) [22].

#### 2.7.3. Target Preparation

The 3D crystal structures of target proteins COX-2 (PDB ID: 5F19) and TNFα (PDB ID: 2AZ5) were retrieved from the RCSB-protein databank. The proteins were prepared in BIOVIA discovery studio v. 21.1.0.20298 (2020) by removing water molecules, steric clashes, and pre-docked ligands. The polar hydrogens and Kollman charges were added.

#### 2.7.4. Molecular Docking

Molecular docking was performed on AutoDock Vina v.4.2.0 [23]. The Lamarckian genetic algorithm was used, and results were analyzed by docking score (Gibbs Free energy). The protein targets COX-2 and TNFα were used as macromolecules. The protein–ligand complex with the lowest RMSD and binding energy from the top 10 poses were selected for molecular dynamics simulation (MDS).

#### 2.7.5. Molecular Dynamic Simulation

The MDS was performed on the GROMACS (2020.4) [24]. The protein–ligand interaction was observed for 100 ns using CHARMM36m forcefield (2020). The trajectory and energy files were written every 10 ps. The production run for simulation was carried out at a constant temperature of 300 K and a pressure of 1 atm (NPT). The MDS results were analyzed based on root mean square deviation (RMSD), the radius of gyration (RoG), and the number and strength of H-bonds. The conformational change of ligand–protein complexes was analyzed on a time scale of 0, 50, and 100 ns.

#### 2.7.6. Binding Free Energy Calculation

The binding free energies (DG) were calculated by the MMGBSA approach. The net DG of the system was determined by finding the difference in DG between the ligand only, the protein only, and their complex, as expressed in the following equation:

ΔG _bind_ = ΔG _complex_ − ΔG _receptor_ − ΔG _ligand_

This DG represents Gibb’s free energy, which is measured via MMGBSA, as shown in the following equation:

ΔG = ΔE _gas_ + ΔG _Solv_ − ΔTS _solute_

The above equation represents the DG calculation, with E _gas_ representing the energy from the molecular mechanics force field and “T” and “S” representing the temperature and entropy of ligand binding, respectively. The E _gas_ term encompasses electrostatic energies, internal energy, and van der Waals interactions.

### 2.8. Statistical Analysis

All data were expressed as mean ± standard deviation (SD). The data from the antioxidant and protein stability assays were analyzed via the nonlinear regression method. The data for paw volume, spleen size, blood CRP, histology scores, and cell viability were analyzed in GraphPad Prism v.8.0.1 by one-way and two-way ANOVA. The level of significance was *p* < 0.05.

## 3. Results

### 3.1. CLHE Phytochemical Screening, Quantification and Characterization

**Figure 2:**
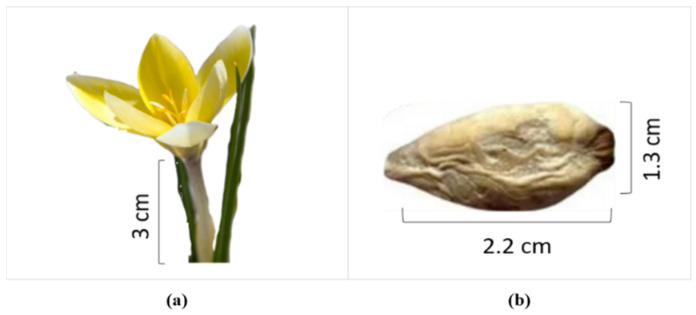
*Colchicum luteum* identification by (**a**) flower and stem, (**b**) corm.

The identification of *C. luteum* was confirmed through its characteristic yellow flowers (Figure 2a), and the corms were selected for extract preparation (Figure 2b). This process yielded a 21% dried extract from 315 g of corms, reflecting efficient extraction of bioactive constituents from the corms. The yield indicates the richness of the corms in potentially active compounds, making them suitable for further biochemical and pharmacological evaluation.

A qualitative analysis of the secondary metabolites present in the CLHE of the corms is summarized in Table 1. The results revealed an abundance of phenols, alongside the presence of alkaloids, anthocyanins, leucoanthocyanin, coumarins, saponins, emodins, sterols, and glycosides. In contrast, tannins, phlobatannins, terpenoids, steroids, and amino acids were absent in the extract. The presence of these bioactive compounds underscores the medicinal potential of the corm extract.

The total phenolic content of CLHE was calculated by analyzing the absorbance of the extract on the standard gallic acid curve. The total phenolic content was 4.91 ± 0.085 mg of GAE/gram of extract. Total flavonoid content was calculated using the rutin calibration curve. The corms of *C. luteum* were estimated to have a flavonoid content of 2.01 ± 0.0424 mg (R^2^ = 0.98).

The GC-MS chromatographic analysis of CLHE separated 600 volatile compounds, among which phenolics, flavonoids, alkaloids, and sequesterpine hydrocarbons were notably abundant, comprising 10.8%, 10%, 8.5%, and 3% of the extract, respectively. From the total pool, select compounds with previously reported medicinal properties were highlighted. For phenolics, 2,3-dihydrobenzoic acid, thymol TBDMS derivative, quinol, and mandelic acid were identified, each known for antioxidant, antimicrobial, and antitumor effects [25,26,27,28]. Alkaloid analysis revealed N-alpha-methylhistamine, 1-(5-fluoro-2-nitrophenyl)piperidine, and tetraponerine T4, compounds linked to neurotherapeutic, anti-inflammatory, and anticancer activities [29,30,31]. Flavonoids such as 2-formyl-9-[β-d-ribofuranosyl]hypoxanthine, 6-chloro-2-cyclohexyl quinazolin-4(3h)-one, and 12-cinnolinedicarboxylic acid were detected, known for their anti-neurodegenerative, sedative, and antimicrobial effects [32,33,34,35]. Additionally, the sequesterpine hydrocarbon ginsenol, with antiviral and antifungal properties [36], was noted. The full list of identified compounds, along with their retention times and peak areas, has been provided in the Supplementary Table S1 for further reference. These selected compounds were discussed to illustrate the therapeutic potential of the CLHE extract.

### 3.2. CLHE Biological Potential

**Figure 3:**
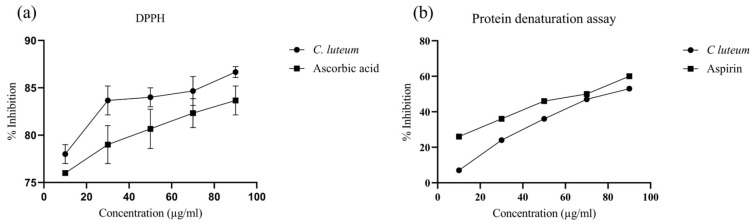
Biological potential of CLHE expressed as (**a**) DPPH assay: The radical scavenging potential of CLHE shows a concentration-dependent increase comparable to ibuprofen. (**b**) Protein denaturation assay: Protein denaturation inhibition activity is significantly increased with extract concentration. CLHE is a significant inhibitor of protein denaturation at 250 µg/mL (*n* = 3) (R^2^ = 0.96).

The plant extract prevents the denaturation of the protein by heat. The percentage of inhibition increases with the increase in concentration, exhibiting extract anti-inflammatory potential. The percentage inhibition at the highest concentration of 550 was 53% ± 0.007 and 60% ± 0.006 for CLHE and aspirin (Figure 3a). IC-_50_ for CLHE was calculated as 97.39 (R^2^ = 0.99), while for aspirin, it was 292.2 (R^2^ = 0.99).

DPPH was utilized to detect the presence of antioxidants in the plant extracts. The higher the percentage of inhibition, the higher the antioxidant properties exhibited by the plant. The percentage of inhibition showed that the plant extract scavenged free radicals in a dose-dependent manner (10–90 µg/mL). The antioxidant capacity of CLHE was recorded as higher than ascorbic acid (Figure 3b). The IC_50_ was for CLHE was calculated as 6.78 (R^2^ = 0.86), while for ascorbic acid, it was 99.85 (R^2^ = 0.80).

### 3.3. Invitro Cytotoxicity Effect of CLHE

**Figure 4:**
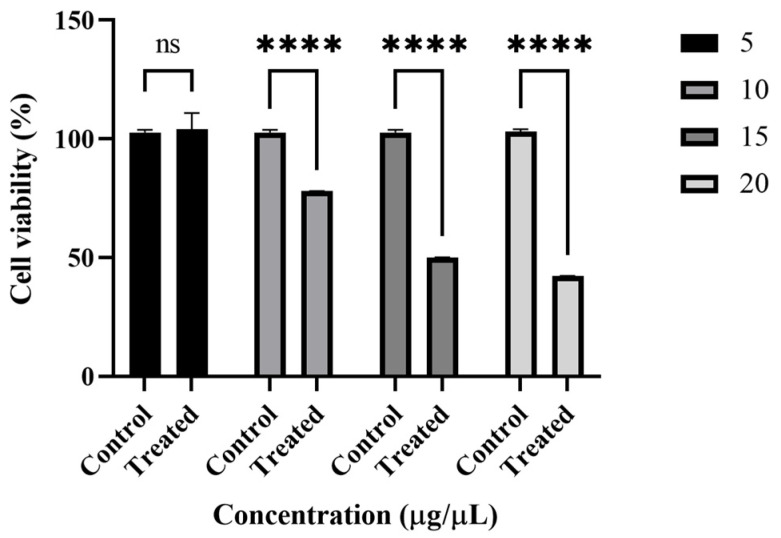
Cytoprotective activity of CLHE expressed as cell viability % in HCEC cells R^2^ = 0.9 Statistical significance was determined by one-way ANOVA, followed by Bonferroni multiple comparison test where ns = non-significant and **** *p* < 0.0001.

The potential cytotoxic effect of CLHE was evaluated on HCEC cell lines via MTT assay for 48 h. CLHE was tested at concentrations ranging from 2 to 20 µg/mL, with results depicted in Figure 4. Cell viability was significantly reduced with increasing concentrations (*p* < 0.0001), with CLHE exhibiting toxicity at doses greater than 10 µg/µL. The concentration of CLHE responsible for a 50% reduction in cell viability (IC50) was calculated to be 78.5 µg/mL for the HCEC cell line, highlighting the significant safety profile of CLHE. Control cells, treated with the same solvent without CLHE, demonstrated 100% viability, providing a baseline for comparison.

### 3.4. In Vivo Acute Toxicity Effect of CLHE

The acute lethal toxicity results show that CLHE does not cause any death in any of the phases. Even at the highest dose of 5000 mg/kg, no death or noticeable physical or behavioral changes were observed, suggesting that the extract is safe for oral administration. Except for increased motor activity in the Dose II group, none showed abnormal physical and behavioral changes (Table 2). Moreover, no significant difference was detected in the creatinine, urea, and total bilirubin levels of mice treated with CLHE, as shown in Table 3.

### 3.5. Effect of CHLE on Paw Swelling and Arthritic Index

**Figure 5:**
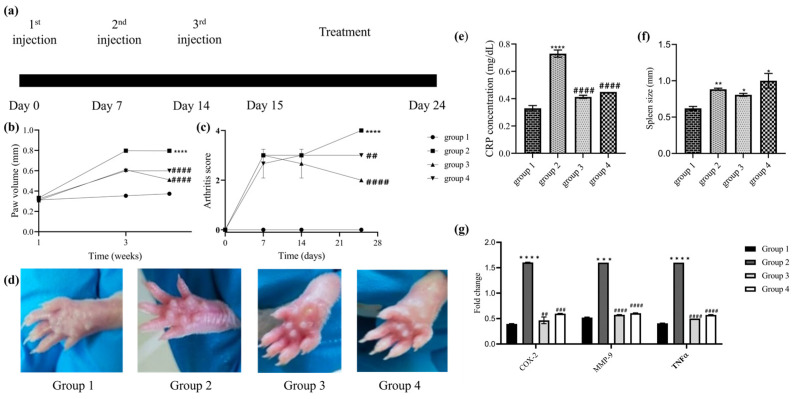
Anti arthritic activity of CLHE. (**a**) Experimental design, (**b**) paw edema, (**c**) arthritic index, (**d**) paw at the end of the experiment, (**e**) blood CRP levels, (**f**) spleen indices, (**g**) expression analysis of COX2, TNFα, and MMP9. Statistical significance was determined by two-way ANOVA or one-way ANOVA, wherever applicable, followed by Bonferroni multiple comparison test where * *p* < 0.01, ** *p* < 0.001, *** *p* = 0.0001 **** *p* < 0.0001 represents control group vs. disease control group and ## *p* < 0.001, ### *p* = 0.0001 and #### *p* < 0.0001 represents disease control group vs. treatment groups.

A CIA model was developed to evaluate the therapeutic effect of *C luteum*. Immunization with CII leads to the development of CIA models with significant paw swelling and inflammation (Figure 5a). There was a significant increase in the paw size from week 1 to 4 (*p* < 0.0001) in group 2 as compared to the healthy control (Figure 5b). A significant thickness in the paws of mice was observed between 1.5 and 2 weeks. The paw volume increased with time in all groups. However, after the treatment, the paw volume significantly decreased for both CLHE and ibuprofen as compared to the arthritic control (*p* < 0.0001).

The arthritis score was determined after regular intervals of 7 days. The scoring ranges from 0 to 4 and is linked to clinical observations. According to this criterion, the mice with maximum scores of 3 and 4 were selected for further experimentation. All three groups showed visible swelling on the entire paw extending to the tarsal joint. Moreover, the selected mice were unable to hold cage wires with the swollen paw, indicating restriction in paw function. The joint swelling and damage were significant on day 7 and boosted substantially for the arthritic control throughout the study (*p* < 0.0001). However, a significant reduction was observed in the treatment groups, i.e., fever clinical signs of inflammation. The extract-treated group showed a greater reduction in arthritic index (*p* < 0.0001) than the standard ibuprofen-treated group (*p* < 0.001) (Figure 5c).

### 3.6. Effect of CHLE on C-Reactive Protein

CLHE reduces CIA-induced inflammation by decreasing the serum levels of CRP. Results showed that the arthritic mice model had elevated levels of CRP as compared to the healthy control (*p* < 0.0001). Treatment with the extract and ibuprofen significantly reduced the level of CRP as compared to the arthritic group *p* < 0.0001, i.e., both ibuprofen and extract showed comparable results in reducing the levels of CRP (Figure 5e).

### 3.7. Effect of CHLE on Spleen Indices

An enlarged spleen is indicative of an inflated immune response and inflammation. To evaluate the severity of arthritis in animal models, spleen indices were conducted. Spleen indices were significantly increased in the CIA model (*p* < 0.001). No significant difference was found between the CLHE- and ibuprofen-treated groups in comparison to the arthritic group. However, spleen indices were significantly more increased for ibuprofen (*p* < 0.0001) than extract (*p* < 0.01) as compared to the healthy mice (Figure 5f).

### 3.8. Effect of CLHE on Inflammatory Biomarkers

The expression of TNFα, COX-2, and MMP-9 genes was analyzed by qPCR. Group 1 showed basal level expression while group 2 showed a significant upregulation of COX2, TNFα, and MMP9 (*p* < 0.0001) compared to the healthy mice. The expressions of COX2, TNFα, and MMP9 significantly decreased in both CLHE-treated and ibuprofen-treated groups. Interestingly both extract and ibuprofen showed comparable results (Figure 5g).

### 3.9. Effect of CLHE on Histopathological Changes

**Figure 6:**
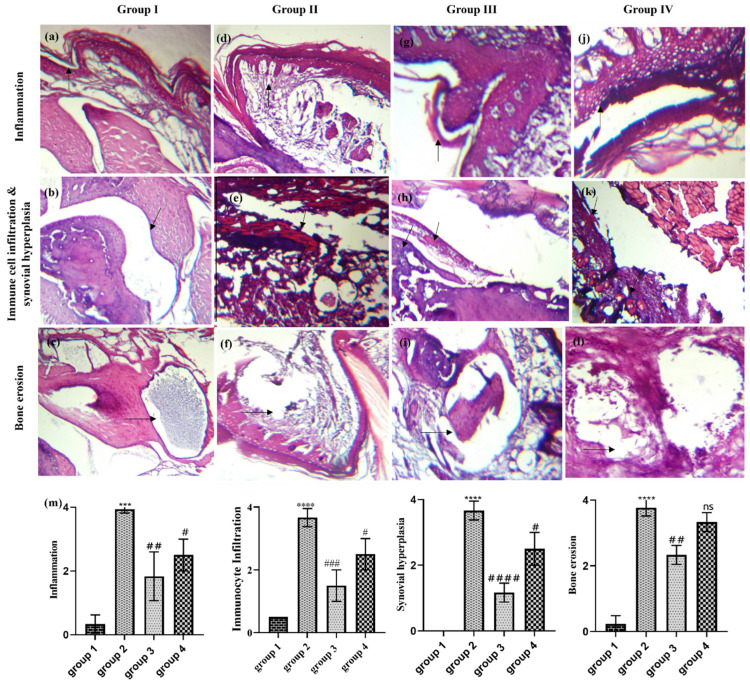
H&E-stained tissue of the tarsal joint of CIA Balb/c mice. (**a**–**c**) Group 1: (**a**) inflammation, (**b**) immune cell infiltration and synovial hyperplasia, (**c**) bone erosion represented by black arrows. (**d**–**f**) Group 2: (**d**) inflammation, (**e**) immune cell infiltration and synovial hyperplasia, (**f**) bone erosion represented by black arrows. (**g**–**i**) Group 3: (**g**) inflammation, (**h**) immune cell infiltration and synovial hyperplasia, (**i**) bone erosion represented by black arrows. (**j**–**l**). Group 4: (**j**) inflammation, (**k**) immune cell infiltration and synovial hyperplasia, (**l**) bone erosion represented by black arrows. Figures were scaled to 100 µm and original magnifications were 20× and 40×. (**m**) Histopathological scoring; statistical significance was determined by one-way ANOVA, followed by Bonferroni multiple comparison test where *** *p* = 0.0001, **** *p* < 0.0001 represents control group vs. disease control group and # *p* < 0.01, ## *p* < 0.001, ### *p* = 0.0001 and #### *p* < 0.0001 represents disease control group vs. treatment groups where ns = non-significant.

Histopathological analysis of the arthritic mice showed visible signs of inflammation, immunocyte infiltration, synovial hyperplasia, and bone erosion. However, treatment with extracts alleviated the arthritic symptoms significantly (Figure 6). Enlarged and effused joints indicate hyperplastic and hypertrophic synovium, which was observed in the arthritic group. The joint enlargement was reduced in the CHLE joint, where relatively smoother membrane boundaries were observed. Treatment with the extract significantly reduced inflammation (*p* < 0.001), immune infiltration (*p* < 0.0001), and synovial hyperplasia (*p* < 0.0001) as compared to ibuprofen (*p* < 0.01). Similarly, in the CLHE-treated group, a significant reduction in the reversal of bone erosion was also recorded (*p* < 0.001), whereas the ibuprofen-treated group showed a non-significant reduction as compared to the arthritic control (Figure 6i–l).

### 3.10. In Silico Analysis of C. luteum Bioactive Compounds

#### 3.10.1. Screening of Bioactive Compounds

**Figure 7:**
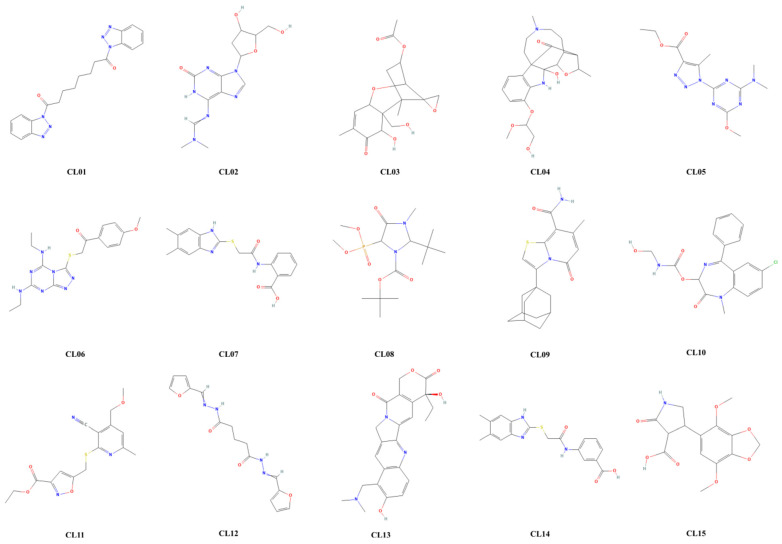
Bioactive natural compounds identified in CHLE through GC-MS from CL01 to CL15.

GCMS analysis identified 600 phytocompounds. Out of the 600 CLHE-derived natural compounds, pharmacokinetic screening identified 15 phytoconstituents (CL01 to CL15) that fulfilled the parameters of absorption, distribution, metabolism, and excretion (Figure 7). Out of these fifteen compounds, CL-1, -4, -5, -10, -11, and -13 presented a Lipinski violation (molecular weight > 350 g/mol). CL-2 presented low GI absorption. CL-3 was identified as a natural toxin. CL-6 was an interfering or “promiscuous” compound with potential off-target effects identified by pan-assay interference structure (PAINS). CL- 7, -8, -9, and -14 presented lower Log S values, rendering them moderately soluble. CL-12 was a chemically reactive and metabolically unstable moiety identified by Brenk alert (Supplementary Table S2). However, compound 15 (CL15) was screened as an ideal candidate for evaluation as it fulfilled the ideal physicochemical profile of absorption, distribution, metabolism, excretion, and toxicity (Table 4).

#### 3.10.2. Toxicity Profiling of Bioactive Compound

**Figure 8:**
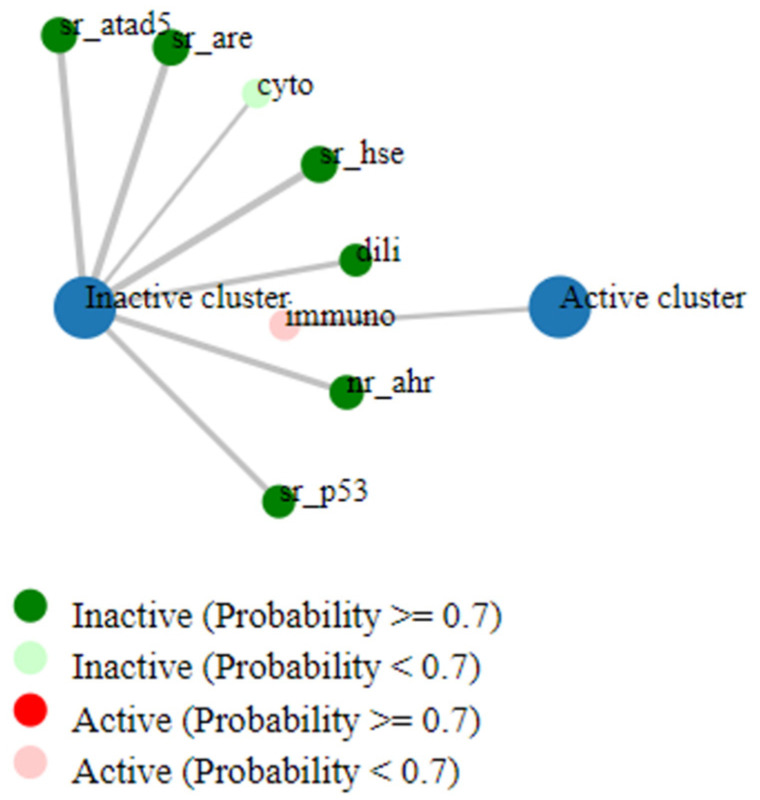
ProTox 3.0 indicating safety of CL15 in all parameters except slight immunotoxicity (*p* = 0.7).

The toxicity analysis of CL15 classified it as non-toxic, placing it within the class IV toxicity category according to the Globally Harmonized System (GHS) for chemical classification and labeling. Compounds in this category are considered non-toxic but may pose a risk if ingested. The predicted lethal oral dose for CL15 was determined to be 450 mg/kg, with a 0.7 probability of causing immunotoxicity (Figure 8).

#### 3.10.3. Molecular Docking

**Figure 9:**
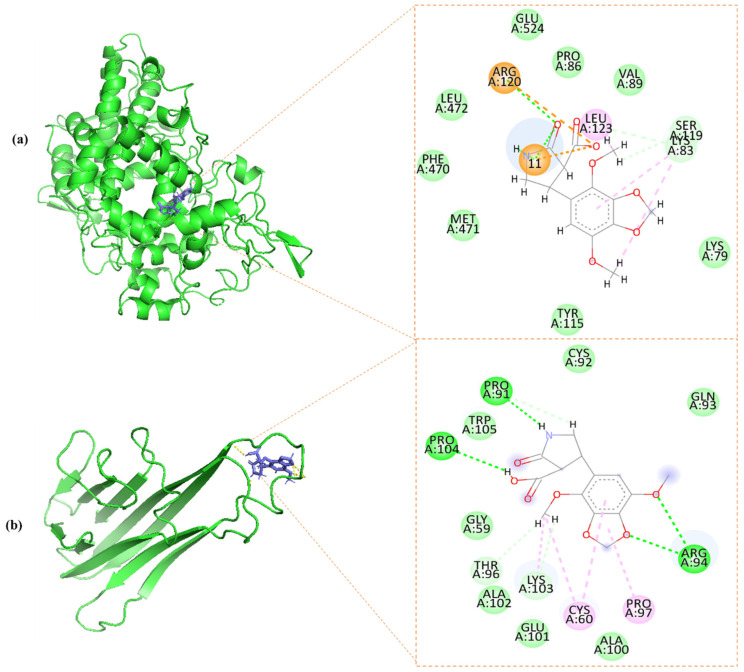
Molecular interaction of CL15 with (**a**) COX-2 and (**b**) TNFα.

Molecular docking analysis revealed a strong binding affinity of CL15 with COX-2 (−12.5 Kcal/mol) and TNFα (−5.8 Kcal/mol). CL15 interacts with COX-2 with four H-bonds at ARG^120^, SER^119,^ and LYS^83^ residues (Figure 9a), while with TNFα, it interacts with seven H-bonds at PRO^91^, ARG^94^, PRO^104^, THR^96^, and LYS^103^ residues (Figure 9b).

#### 3.10.4. Molecular Dynamics Simulation

**Figure 10:**
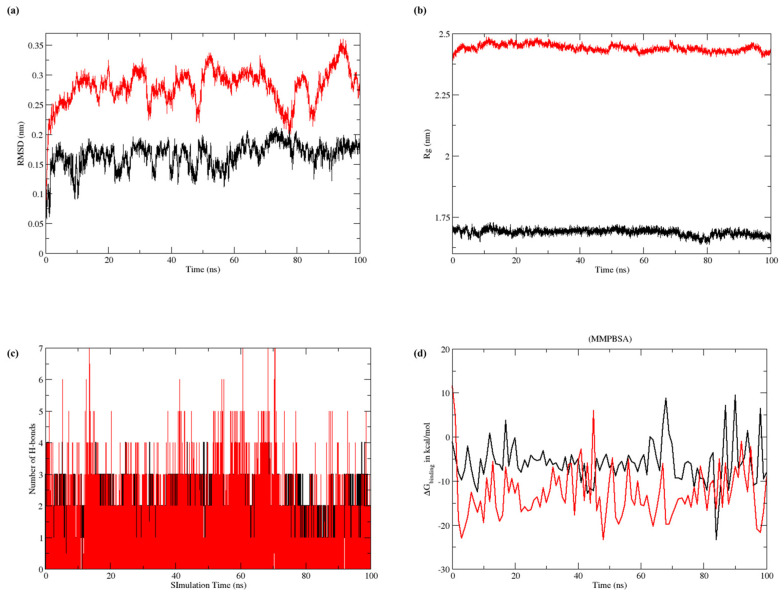
Molecular dynamics simulation graphically representing (**a**) RMSD, (**b**) RoG, (**c**) H-bond, and (**d**) MMGBPSA, where red represents complex 1 and black represents complex 2.

Protein–ligand complexes of CL15 with COX2 (complex 1) and TNF α (complex 2) were further evaluated for 100 ns simulation. For complex 1, the mean RMSD was 0.28 ± 0.03 nm, with a rising trend after 30 ns. Complex 1 underwent a conformational change from 70 to 90 ns; however, the changes remained within the acceptable values (0.12 nm). For complex 2, the average RMSD was 0.16 ± 0.02 nm, with minimum fluctuation throughout the simulation. The complex showed minor fluctuations at the beginning of the simulation; however, the complex remained stable after 7 ns (Figure 10a).

The RoG for complex 1 was 2.44 ± 0.01 nm, and no significant changes were observed in the compactness of the complex. The RoG for complex 2 was 1.68 ± 0.01 nm. A slight decrease in complex 2 radii was observed at 80 ns; however, the compactness of the complex was not affected (Figure 10b).

The H-bond strength was calculated as the average number of bonds within the complexes. Complex 1 shows consistent bond formation throughout the simulation, valued at 1.70 ± 0.67. The density of the graph indicates the strength of H-bonds. For complex 2, consistent, but low-strength, H-bonds were observed throughout the simulation after 5 ns. The average number of H-bonds in complex 2 was 1.66 ± 0.5 (Figure 10c).

Binding free energies were estimated by employing MMGBSA methods to better understand the complexes’ binding abilities with target proteins. Stable complexes were generated because all the binding interactions were energetically favorable, and Table 5 displays the results of these experiments. The net energy in MMGBSA for COX2-CL15 and TNFα-CL15 were calculated as −12.72 (5.98 ± 0.59) and −14.42 (8.12 ± 0.25) kcal/mol, respectively. Moreover, the ∆G graph remained stable throughout the simulation (Figure 10d).

**Figure 11:**
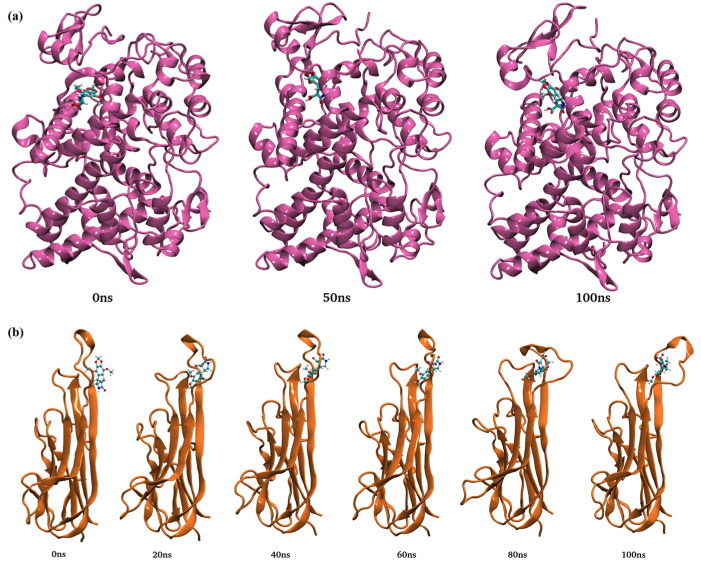
Conformational change and structural rotation of (**a**) complex 1 and (**b**) complex 2.

The conformation of the complexes was observed from 0 to 100 ns. An *N*-terminal shift was observed in complex 1; however, CL15 remained stably bound to the substrate-binding domain of COX-2 (Figure 11a). Conformation changes of complex 2 also showed the stable binding of CL15 with the binding pocket of TNFα (Figure 11b).

## 4. Discussion

RA is a chronic autoimmune disorder marked by severe pain, inflammation, and progressive joint deterioration. Its global incidence is rising rapidly, yet current treatment options remain limited. NSAIDs, commonly used as the first line of treatment, are associated with significant side effects such as gastrointestinal bleeding and cardiovascular risks. Advanced therapies, including biologics, are costly, target only specific pathways, and also carry the risk of adverse effects [37]. As a result, there is a growing shift toward alternative therapeutic strategies to address these limitations. Medicinal plants have long been explored as potential remedies for RA. However, their widespread use, often regarded as safe, is frequently based on incomplete knowledge of their chemical makeup and a lack of understanding about proper dosing and toxicity risks. To identify viable drug candidates, it is crucial to thoroughly examine the chemical composition, safe dosage levels, and toxicity profiles of these plants. This study aims to evaluate the safety and anti-inflammatory effects of *C. luteum* while identifying safer and more effective bioactive compounds for RA treatment through in vitro, in vivo, and in silico methodologies.

*C. luteum* extract was analyzed for secondary metabolites, revealing the presence of pharmacologically important compounds such as flavonoids, phenols, and emodin (Table 1). Nearly 20% of the metabolites identified through GC-MS analysis were phenolics and flavonoids, indicating a strong antioxidant profile. Additionally, 8.5% of the identified compounds were alkaloids and their derivatives. Notably, colchicine was not detected in the extract, which is significant given its high toxicity that limits its therapeutic use, particularly as an anti-tumor agent [38]. This absence suggests a safer pharmacological profile for CLHE, enhancing its therapeutic potential while mitigating the risks associated with colchicine itself, thereby making CLHE a more favorable option for therapeutic applications.

Both phenols and flavonoids play a key role in providing antioxidant and anti-inflammatory effects [39]. CLHE demonstrated substantial concentrations of these phytochemicals, which aligned with its biological activity in antioxidant and anti-inflammatory assays. The DPPH assay, used in this study, specifically measures the radical scavenging activity of the extract. While the assay does not directly quantify antioxidant capacity, radical scavenging is a key mechanism through which antioxidants function, as it neutralizes free radicals that contribute to oxidative stress. Thus, the DPPH assay provides valuable insight into the antioxidant potential of CLHE. Previous studies have validated the use of DPPH as an indicator of antioxidant properties in biological samples [40], supporting its relevance to the current study.

Notably, CLHE demonstrated superior effectiveness in inhibiting protein denaturation compared to ibuprofen, suggesting its better potential to protect cells from inflammation-induced damage. Furthermore, its enhanced ability to scavenge and stabilize free radicals beyond that of the standard control highlights its promising role in reducing oxidative stress and preventing cellular harm.

In drug design and development, toxicology is as crucial as efficacy. The cytotoxicity of CLHE was assessed using the HCEC cell line, which originates from a healthy adult’s colonic biopsy and is widely used as an in vitro model for studying intestinal absorption and toxicology studies [41]. After 48 h of treatment with CLHE, 48% cell viability was observed at the highest concentration (20 μg/mL) (Figure 5a). These findings suggest that CLHE is non-toxic to healthy organs and gastrointestinal tissues. The acute toxicity assessment, using the Enegide method, provided a reliable and reproducible approach with minimal animal use, reducing the need for excessive animal testing [42]. Oral administration of CLHE did not result in any behavioral or neurological abnormalities, and its high LD50 (5000 mg/kg) confirms its safety at elevated doses. Additionally, fasting for 12 h prior to dosing ensures no interference from previous feed intake (Table 2). The lack of significant changes in serum urea, bilirubin, or creatinine levels, compared to healthy controls, further supports the non-toxic profile of CLHE [43]. Nasir et al. previously conducted an acute toxicity study of *C. luteum* on mice, with a maximum dose of 2000 mg/kg, reporting no adverse effects [44]. Our findings extend this, demonstrating that even at a dose of 5000 mg/kg, CLHE remains safe, validating the earlier study.

Once the safety of CHLE was established, its anti-inflammatory potential was evaluated in a CIA mice model, using ibuprofen as a reference NSAID. Previous studies have explored the effects of *C. luteum* in arthritis models, but its comparison against any NSAID has not been established [45]. In this study, oral administration of CLHE resulted in a significant reduction in both systemic and localized inflammation in the arthritic mice. Peripheral edema, a key indicator of inflammation, directly correlates with disease severity and progression [46]. In the post-treatment groups, the reduction in paw volume in group 3 highlights the ability of CLHE to effectively reduce edema. Interestingly, CLHE demonstrated a greater reduction in arthritic scores compared to ibuprofen, indicating its superior efficacy in reducing both inflammation and arthritis-related symptoms.

At the organ level, spleen size is an important indicator of inflammation. During inflammation, the spleen is enlarged to cater to the increased circulation of cytokines [47]. In the disease model, the spleen size increased to maximum of 1 mm, which was significantly reduced in the post-treatment groups. Notably, spleen indices were markedly higher in the ibuprofen-treated group (*p* < 0.0001) than in the CLHE-treated group (*p* < 0.01), further emphasizing CLHE’s enhanced anti-inflammatory potency. CRP is a systemic inflammatory marker whose concentration increases in response to tissue damage and inflammation [48]. While CRP levels were significantly elevated during the inflammatory phase, the post-treatment groups showed marked reductions in CRP concentrations. The observed decrease in CRP in groups 3 and 4 suggests that the anti-inflammatory effects of CLHE are comparable to those of ibuprofen.

Further assessment of the anti-inflammatory effects of CLHE was conducted via the expression of key inflammation-related genes. CLHE significantly downregulated the expression of COX-2, TNFα, and MMP-9, all of which play critical roles in RA-induced inflammation [49]. TNFα, produced by T-lymphocytes and macrophages, is involved in joint damage and immune cell activation [50]. Its upregulation leads to increased COX-2 expression, which promotes prostaglandin synthesis. MMP-9, activated by both TNFα and COX-2, is a key enzyme responsible for cartilage degradation and bone erosion [51,52]. The comparable reduction of COX-2, TNFα, and MMP-9 by both CLHE and ibuprofen suggests that CLHE effectively reduces immune cell activation and offers protection against joint damage. Similar results were confirmed by histopathological analysis of the paw, where the immunomodulatory potential of the CLHE-treated group showed reversal of bone erosion and alleviation of arthritic-induced inflammation, immunocyte infiltration, and synovial hyperplasia to a greater degree than ibuprofen. Therefore, CHLE can be considered as a therapeutic with a comparatively safer profile as compared to NSAIDs with severe cytotoxic side effects.

The use of in silico studies to evaluate the toxicity of drug or plant-based compounds has become increasingly popular due to their cost- and time-effectiveness [53]. Following GC–MS analysis, an in silico approach is commonly employed for the initial screening of phytochemicals from medicinal plants [54]. To explore the mechanism of action for safer, non-toxic bioactive compounds, the library of CLHE constituents identified via GC-MS was subjected to ADMET screening. This computational tool assesses the pharmacokinetic profiles of compounds, focusing on absorption, distribution, metabolism, and excretion, which are direct predictors of biological activity. Among the natural compounds derived from CLHE, fifteen met the ADMET criteria, but only one compound, CL15, satisfied all the parameters for drug-likeness.

Further toxicity analysis of CL15, conducted through the Protox 3.0 tool, revealed its safety across various toxicological endpoints, including hepatic, neurological, cardiac, and respiratory systems, characterizing it as non-toxic. To evaluate its potential as an alternative to NSAIDs, CL15 was docked with COX-2 and TNFα, two key targets in inflammation. COX-2 is the primary target for NSAIDs in reducing inflammatory symptoms, while TNFα induces COX-2 expression [37]. CL15 exhibited significant pharmacological and anti-inflammatory potential through its strong binding affinity with both COX-2 and TNFα, which was further validated by stable simulations of these complexes. In the case of TNFα, CL15 binds at positions 91 and 94, which is closer to a structurally important residue at position 90. Position 90 is a key residue that is involved in maintaining the TNFα loop structure and contributes to receptor binding activity, as shown in amino acid substitution models [55]. Hydrogen bond formation at residues 91, 94, 96, and 104 with sufficient stability within the protein structure potentially exerts anti-inflammatory effects, as observed in the in vivo evaluations. The interaction between CL15 and TNFα remained stable throughout the simulation, suggesting that CL15 may modulate TNFα activity effectively. Similarly, in the case of COX-2, CL15 demonstrated a very stable interaction throughout the simulation. Moreover, low ΔG for both the complex suggested high stability in the molecular environment and minimal conformational changes over the 0–100 ns MD period.

In COX-2, ARG^120^ is one of the three conserved residues within the active site, responsible for binding the physiological substrate, arachidonic acid, as well as NSAIDs [56]. CL15 binds to ARG^120^ through both covalent (H-bond) and ionic (salt bridge) bonds (Figure 9a). These salt bridges serve as “molecular clips” that stabilize the conformation of the protein–ligand complex, a critical factor in rational drug design [57]. This dual interaction, combined with an additional H-bond at SER^119^, allows CL15 to occupy much of the COX-2 active site, consistent with the number and strength of H-bonds. Notably, SER^119^, located at the entrance of the active site, is unique to COX-2, as COX-1 has a VAL^119^ residue instead. The targeted interaction of CL15 with SER^119^ contributes to its selective inhibition of COX-2 [56]. Thus, this study suggests that CL15 has the potential to overcome the limitations of current RA treatments. NSAIDs, which are commonly used as the first line of therapy, carry significant risks, while biologics, though effective in targeting TNFα, are expensive and often cause side effects. CL15, with its targeting of both COX-2 and TNFα, offers a more comprehensive and safer alternative; however, further wet lab experiments are needed to quantify and validate its therapeutic potential.

## 5. Conclusions

Our study demonstrates the therapeutic potential of CLHE in the management of RA. Through a combination of in vitro, in vivo, and computational analyses, we identified CLHE’s anti-inflammatory and antioxidant properties, along with its safety profile at high doses. Specifically, we found that CLHE significantly reduced inflammation and arthritic symptoms in a collagen-induced arthritis model without inducing toxicity. The identification of CL15 as a selective inhibitor of COX-2 and TNFα, key mediators in RA, further underscores the therapeutic promise of *Colchicum luteum*. These findings suggest that CLHE could serve as a safer alternative to NSAIDs, offering multi-targeted efficacy for RA management. However, further clinical studies are required to validate its effectiveness in humans.

## Figures and Tables

**Table 1:** Phytochemical constituents of *C. luteum* corms extract: (+) present, ++ (abundant), and (-) absent.

S. No.	Secondary Metabolites	*C. luteum* Hydroalcoholic Extract
1	Alkaloids	+
2	Phenols	++
3	Flavonoids	+
4	Anthocyanins	+
5	Leucoanthocyanins	+
6	Tannins	-
7	Phlobatannins	-
8	Coumarins	+
9	Terpenoids	-
10	Steroids	-
11	Saponins	+
12	Emodins	+
13	Amino acids	-
14	Sterols	+
15	Glycosides	+

**Table 2:** Acute toxicity evaluation of CLHE.

Signs of Acute Toxicity	Dose I: 800 mg/kg	Dose II: 2000 mg/kg	Dose III: 5000 mg/kg
Sedation	-	-	-
Respiratory distress	-	-	-
Salivation	-	-	-
Hyperesthesia	-	-	-
Diarrhea	-	-	-
Blanching	-	-	-
Increased motor activity	-	+	-
Writhing/twisting	-	-	-
Straub reaction	-	-	-
Tremors	-	-	-
Arching and rolling	-	-	-
Tonic convulsions	-	-	-
Tonic extension	-	-	-
Lacrimation	-	-	-
Cyanosis	-	-	-
Number of deaths	0	0	0

+ indicates presence of symptoms and - indicates absence of symptoms

**Table 3:** Creatinine, urea, and total bilirubin levels of CLHE treated mice.

Test Name	Control	CLHE
T. Bilirubin	0.6	0.5
Urea	36	45
Creatinine	0.33	0.20

**Table 4:** Pharmacokinetic profile of CL15 compared to the accepted values of ADMET.

PK Category	Parameters	CL15	Accepted Value
Absorption	HIA	0.1	0 ≥ 30% 1 ≤ 30%
Distribution	Volume distribution	0.247	0.04–20 L/kg
	BBB penetration	0.28	The value corresponds to the probability
Metabolism	CYP1A2	0.496	>0.0
	CYP2C19	0.214	>0.0
	CYP2D6	0.005	>0.0
	CYP3A4	0.100	>0.0
Excretion	Clearance	1.34	5–15 mL/min/kg
	Half-life	1.58	0–3 h
Drug-likeness	Molecular weight	309.08	100–600
	Polar surface area	103.32	0–140
	H-bond donors	2	0–7
	H-bond acceptors	8	0–12
	No. of rotatable bonds	4	0–11

**Table 5:** Binding free energies (Kcal/mol) and the individual energetic terms for the systems using the MMPBSA method.

Parameters	COX-2	TNFα
	CL15 Avg. Binding Energy (Std. Dev ± Std. Err. of Mean	CL15 avg. Binding Energy (Std. Dev ± Std. Err. of Mean
Van der Waals (EVDW)	31.91 (2.99 ± 0.29)	27.11 (8.68 ± 0.27)
Electrostatic (EEL)	11.07 (8.19 ± 0.81)	15.94 (8.63 ± 0.27)
Polar (EPB)	33.89 (7.62 ± 0.75)	31.57 (9.36 ± 0.29)
Non-polar (ENPOLAR)	−3.62 (0.13 ± 0.01)	−2.94 (0.44 ± 0.01)
EDISPER	0.00	0.00
Delta G (gas)	−42.98 (9.63 ± 0.95)	−43.06 (13.95 ± 0.44)
Delta G (sol)	30.26 (7.56 ± 0.75)	28.63 (9.02 ± 0.28)
Delta Total	−12.72 (5.98 ± 0.59)	−14.42 (8.12 ± 0.25)

## Data Availability

All data generated or analyzed during this study are included in this article and Supplementary Information Files.

## Supplementary Materials

The following supporting information can be downloaded at: https://www.mdpi.com/article/10.3390/nu16234020/s1, Table S1: GC-MS of C. luteum extract; Table S2: ADMET screening data of CL01-CL15.

## Abbreviations

Arg	Arginine
CLHE	*Colchicum luteum* hydroethanolic extract
COX-2	Cyclooxygenase 2
CRP	C-reactive protein
DMARDs	Disease-modifying anti-rheumatic drugs
DMEM	Dulbecco’s modified Eagle medium
DPPH	2,2-diphenyl-1-picrylhydrazyl
FBS	Fetal bovine serum
GAPDH	Glyceraldehyde-3-phosphate dehydrogenase
GC-MS	Gas chromatography-mass spectrometry
GHS	Globally harmonized system
H&E	Hematoxylin and eosin
H-bond	Hydrogen bond
HCEC	Human colon epithelial cells
IC_50_	Half maximal inhibitory concentration
LD_50_	Lethal dose 50%
MDS	Molecular dynamics simulation
MMP-9	Matrix metalloproteinase 9
MTT	3-(4,5-dimethylthiazolyl-2)-2,5-diphenyltetrazolium bromide
NSAIDs	Non-steroidal anti-inflammatory drugs
Pro	Proline
qPCR	Quantitative polymerase chain reaction
RA	Rheumatoid arthritis
RMSD	Root mean square deviation
RMSF	Root mean square fluctuation
RNA	Ribose nucleic acid
RoG	Radius of gyration
Ser	Serine
TNFα	Tumor necrosis factor-alpha
UV-vis	Ultraviolet-visible
